# Post-Application Field Persistence and Efficacy of *Cordyceps javanica* against *Bemisia tabaci*

**DOI:** 10.3390/jof9080827

**Published:** 2023-08-05

**Authors:** Shaohui Wu, Michael D. Toews, Robert W. Behle, Apurba K. Barman, Alton N. Sparks, Alvin M. Simmons, David I. Shapiro-Ilan

**Affiliations:** 1 Department of Entomology, University of Georgia, 2360 Rainwater Road, Tifton, GA 31793, USA; wu.6229@osu.edu (S.W.); abarman@uga.edu (A.K.B.); asparks@uga.edu (A.N.S.); 2 National Center for Agricultural Utilization Research, USDA-ARS, 1815 N. University St., Peoria, IL 61604, USA; robert.behle@usda.gov; 3 U.S. Vegetable Laboratory, USDA-ARS, 2700 Savannah Highway, Charleston, SC 29414, USA; alvin.simmons@usda.gov; 4 SE Fruit and Tree Nut Research Unit, USDA-ARS, 21 Dunbar Road, Byron, GA 31008, USA

**Keywords:** entomopathogenic fungus, epizootic, field persistence, field efficacy, whitefly

## Abstract

Previously, *Cordyceps javanica* Wf GA17, a causing agent of whitefly epizootics in southern Georgia, demonstrated superior temperature tolerance and higher virulence against the whitefly *Bemisia tabaci* than commercial strains in the laboratory. The post-application persistence and efficacy of this fungus against *B. tabaci* were compared with that of the commercially available *C. javanica* Apopka97 strain over a two-year field study in cotton and vegetable crops. When blastospores of both strains were applied alone, whitefly populations were not effectively suppressed. Thus, JMS stylet oil was added to fungal treatments for enhancing efficacy and persistence. For 0-day samples, all fungal treatments caused similar but significant levels of immature mortality regardless of fungal strain, propagule form (conidia vs. blastospores), and application method (alone or mixed with JMS). In follow-up samplings, Wf GA17 blastospores + JMS achieved higher control levels than other treatments in some trials, but the efficacy did not last long. The JMS oil alone caused significant mortality and suppressed whiteflies. Over 90% of spores lost viability 24 h after treatment in all fungal treatments. Across evaluation times, there was no difference between the two fungal strains (conidia or blastospores, alone or combined with JMS), but conidia persisted better than blastospores for both strains. Overall, the field persistence and efficacy of *C. javanica* did not last long; therefore, improved delivery methods and formulations are needed for enhancement.

## 1. Introduction

Entomopathogenic fungi (EPF) naturally infect insects and some EPF strains have been commercially produced as biopesticides for managing insect pests [1,2]. Their field persistence and efficacy may be affected by various abiotic and biotic factors, such as solar radiation, temperature, humidity/moisture levels, surface chemistry, and phylloplane microbiota [2,3,4,5]. However, widespread infection by EPF may occur under favorable conditions, e.g., high humidity, mild temperature, frequent rainfalls, and high host densities, which promote horizontal transmissions in pest populations, leading to epizootics [2]. In 2017, a naturally occurring epizootic among whiteflies was observed in southern Georgia where high levels of whitefly infestations were noted in cotton and vegetable production [6]. The fungus was isolated and identified as a new strain of *Cordyceps javanica* (Wf GA17 strain) [7,8]. This new strain demonstrated high virulence to the sweetpotato whitefly, *Bemisia tabaci* (Gennadius) MEAM1 (Middle East-Asia Minor 1, also known as biotype B) (Hemiptera: Aleyrodidae), and caused higher whitefly mortality and infection levels than commercial strains *C. javanica* Apopka 97 (formerly *C. fumosorosea* (Wize) Kepler, B. Shrestha and Spatafora Apopka 97), *Metarhizium brunneum* Petch strain F52, and *Beauveria bassiana* (Bals.-Criv.) Vuill. strain GHA [7]. In addition, this strain was more tolerant to extreme temperatures than *C. javanica* Apopka 97 [9]. These studies suggest great potential for using *C. javanica* Wf GA17 as biopesticides in pest management, especially against whiteflies in field applications.

The whitefly, *B. tabaci,* infests a variety of crops (such as vegetables, ornamentals, and cotton) and may cause significant crop losses at high population densities [10,11]. The adults normally lay eggs on the leaf undersides; the eggs hatch into tiny crawlers that settle at a feeding location and develop through four immature stages (the fourth instar is also referred to pupa) before emerging into adults. Both adult and immature whiteflies feed on the plant phloem, reduces plant vigor and stunts the growth; meanwhile, it secrets honeydew that causes sooty mold on plant leaves and fruits, affecting crop growth and values. In addition, secondary damage may occur when whiteflies vector plant viruses, which can be more damaging than the direct feeding [12,13]. Whiteflies have been primarily managed with synthetic insecticides but have developed widespread resistance to many commonly used insecticides [14,15]. Under these circumstances, EPF has been considered as a viable solution to combat the resistance issues in managing whiteflies. Various species of EPF have been used for biological control of *B. tabaci* and mostly belong to Hyphomycetes, such as *B. bassiana*, *C. javanica* (formerly *Isaria fumosorosea* or *Paecilomyces fumosorosea*), *Akanthomyces lecanii* (Zimm.) Spatafora, Kepler and B. Shrestha (formerly *Lecanicillium lecanii* or *Verticillium lecanii*) [1,16]. In particular, whiteflies are on the insecticide label of several fungal biopesticides, including NOFLY^™^ and PFR-97^™^ (*C. javanica*) and BotaniGard^®^, BoteGHA^®^ and Velifer^®^ (*B. bassiana*), available in the United States [17].

The interest of using *C. javanica* in whitefly control started in the 1990s, when the strain Apopka97 was isolated and tested for its high virulence to *B. tabaci* and other pests [18,19,20]. Studies on the use of *C. javanica* for controlling *B. tabaci* has been reviewed by Faria and Wraight [16], Lacey et al. [21] and Zimmermann [22]. Many research on the efficacy of *C. javanica* against *B. tabaci* has been conducted in laboratory and greenhouse conditions except a few field studies (e.g., [23,24,25,26]), and data on field efficacy need to be expanded. In addition, the selection of isolates is a key consideration for managing *B. tabaci* with EPF [16]. Given the higher efficacy and environmental tolerance of *C. javanica* Wf GA17 compared to the commercial strain Apopka 97 (in PFR-97^™^) in previous studies [7,9], a potential advantage of the new strain may exist but needs to be verified under field conditions. Hence, the objective of this study was to evaluate the efficacy of *C. javanica* Wf GA17 blastospores for control of whiteflies and assess the post-application persistence under natural field conditions. This research complements previous laboratory studies and further explores the potential use of this new fungal strain in whitefly management, which will assist in directing future research to optimize field performance.

## 2. Materials and Methods

### 2.1. Preparation of Fungal Materials

Blastospores of *C. javanica* Wf GA17 and Apopka97 were grown in 100 mL of liquid culture media containing 1 × 10^5^ conidia/mL in a 250-mL baffled Erlenmeyer flask for four days using a rotary shaker incubator (INNOVA 4000, New Brunswick Scientific, Edison, NJ, USA) set at 28 °C and 350 rpm. Fungal cultures were sieved using a 120-mesh screen to removal fungal hyphae. Skim milk powder (Non-Fat Instant Dry Milk, Great Value, Walmart, Bentonville, AR, USA) was added to screened cultures at 10% *w*/*w* [27]. Cultures were then spray dried using a Niro spray drier (Columbia, MD, USA) with a spinning disk atomizer, at ~85 °C inlet temperature, 47–51 °C outlet temperature, and 5.7 bar wheel atomizer pressure. Blastospore concentrations per gram of product were determined with a hemocytometer under 400× magnification. Blastospore germination percentages were determined using a 6 h liquid culture germination assay [28]. Fungal blastospores were stored under refrigeration (~5 °C) until use.

### 2.2. Field Efficacy in 2019

The field efficacy of blastospores of *C. javanica* Wf GA17 versus Apopka97 against whiteflies was tested in a cotton field (31.5095, −83.6472) at the University of Georgia, Tifton, GA, USA. The treatments included *C. javanica* Wf GA17 blastospores, *C. javanica* Apopka97 blastospores, and an untreated control. Each treatment was replicated in four blocks using a randomized complete block design, and the test was conducted twice. Trial 1 was carried out on 12 August 2019. Fungal blastospores were mixed with water and sprayed at 2.5 × 10^12^ viable blastospores/ha in the spray volume of 168 L/ha using a CO_2_ backpack sprayer equipped with four hollow cone nozzles (ConeJet TXVS-6) at the pressure of 276 kPa. The application was made between 11:30 a.m. and 12:30 p.m. when the weather condition was sunny. The plot size was 2 m width by 2 m length, and there was a 10 m barrier between adjacent plots. After application, a field cage was placed onto each plot to prevent whitefly adults migrating to adjacent plots. The 5th true leaf (counting from top) was taken to count the number of live and dead whitefly immatures and adults at 0, 7, 14 and 21 days after treatment (DAT), and five leaves were randomly sampled from each plot. Percent fungal infection was also evaluated by examining the morphological characteristics, followed by molecular identification using the same procedure for identifying *C. javanica* Wf GA17 [7]. Trial 2 was conducted on 6 September 2019 in a different region of the same cotton field using the same treatments and procedures except that the application rate was doubled to 5 × 10^12^ viable blastospores/ha. During the first and second trials, the average daily air temperature ranged 24–29 and 23–29 °C (maximum observed temperatures were 26–37 and 29–37 °C; minimum observed temperatures were 19–24 and 16–23 °C); the daily R.H. ranged 67–93% and 58–79% on average (maximum 92–99% and 83–99%; minimum 34–81% and 20–51%); the daily total solar radiation was 6–24 and 15–23 MJ/m^2^; there was 133.6 and 0.1 mm of accumulated rainfall, respectively.

### 2.3. Field Efficacy and Post-Application Persistence in 2020

In 2020, a horticultural oil, JMS^®^ stylet oil was added to blastospores to improve the control efficacy against whiteflies. Trial 1 was conducted in an irrigated cotton field (31.5261, −83.5464) at the University of Georgia (Tifton, GA, USA) at the stage of four nodes above white flower on 21 August 2020. There were six treatments: control, JMS oil alone, *C. javanica* Wf GA17 alone, *C. javanica* Wf GA17 plus JMS oil, *C. javanica* Apopka97 alone, and *C. javanica* Apopka97 plus JMS oil. Fungi *C. javanica* Wf GA17 and Apopka97 were applied at 5 × 10^12^ viable blastospores/ha (2.94 ×10^7^ viable spores/mL); the JMS oil was used at 3%. Applications were made at 7 p.m. Treatments were sprayed in 168 L/ha using ConeJet TXVS-6 nozzles at 276 kPa. There were four treatment blocks, and each plot was consisted of 5.5 m width by 6.1 m length, and there was a 10 m buffer between adjacent plots. Five leaves at the 5th true leaf position were randomly taken from each plot at 0, 7, 14, 21 and 28 DAT. Live whitefly counts, mortality and percent mycosis were evaluated. For samples collected immediately after application (0 DAT), leaves were made into leaf discs placed onto 60-mm Petri dishes filled with 2% water agar to incubate fungal growth on whitefly immatures at 25 °C (light 14 h: dark 10 h), and whitely immature mortality and mycosis levels were assessed after 5 and 7 days of incubation. During the field test, the average daily temperature was between 22 and 29 °C, with the maximum between 24–35 °C and the minimum between 20 and 25 °C; there were 14 out of 28 days with rain, accumulating 178 mm of total rain; the R.H. ranged from a minimum of 29–91% to a maximum of 84–100% (average 66–97%); and the total daily solar radiation ranged 1–24 MJ/m^2^.

Trial 2 was carried out in a cotton field (31.5261, −83.5464) at Fort Valley State University (Fort Valley, GA, USA) at three nodes above white flower stage on 6 October 2020. In addition to the six treatments used in the previous trial, conidia of *C. javanica* Wf GA17 and Apopka97 (without JMS oil) were added. All fungal treatments were applied at 5 × 10^12^ viable spores/ha, and JMS oil applied at 3% at 10 a.m. The plot size and design were similar to the first trial, and the experimental procedures were similar except that eight leaves were sampled from each plot instead of five. Similar to the previous test, efficacy against whiteflies was evaluated at 0, 7, 14, 21 and 28 DAT. Additional samplings were taken at 0, 24, 48 and 72 h after application to evaluate fungal persistence post-application. For this purpose, a leaf disc (dia. 1.7 cm; area 2.27 cm^2^) was punctured from each leaf, and a total of eight leaf discs from eight leaves were used to determine fungal viability in each plot at each time. Each leaf disc was rinsed with approximately 1 mL of sterile 0.05% Silwet on each side, and the rinses of eight leaf discs from upper and lower leaf surfaces were collected separately. The collected rinses were diluted up to 10 times, and 0.1 mL of original or diluted suspension was spread onto a PDAY dodine plate (100-mm), which was incubated at 25 °C (light 14 h: dark 10 h) for 4 days to count the number of colony forming units (CFU). The number of CFUs per cm^2^ was evaluated for each plot over time. During the test (4 weeks), the average daily temperature ranged 9–24 °C (minimum 0–21 °C; maximum 17–31 °C); daily R.H. was 39–93% (minimum 19–75%; maximum 87–100%); there were 12 rainy days that produced 36 mm of total rain; the daily solar radiation was 7–17 MJ/m^2^.

Trial 3 was implemented in a snap bean field (31.5261, −83.5464) infested with whiteflies on a research farm at University of Georgia (Tifton, GA, USA) on 30 October 2020. The eight treatments and experimental procedures were the same as the second trial, except that the fungal rate was doubled to 1 × 10^13^ viable spores/ha (5.88 × 10^7^ viable spores/mL), and the plot size was 3 m width by 6 m length, with 3 m by 4 m buffer between adjacent plots. Treatments were sprayed at 9 a.m. Five leaves were sampled at 0, 7, 14 and 21 DAT (28 DAT sampling was not made due to frost) to assess live whitefly immature counts, whitefly mortality and mycosis levels for control efficacy. Additional samplings were made at 0, 6, 24, 48 and 72 h after application to check the post-application persistence of fungal treatments. During the 3-week period, the average air temperature was 10–25 °C (3–23 °C low and 16–30 °C high); the R.H. ranged 12–91% low and 71–100% high, with a daily average of 40–95%; six rainy days produced only 10 mm of rainfall in total; the daily solar radiation was 7–17 MJ/m^2^.

### 2.4. Data Analysis

All data were analyzed using the software SAS version 9.4 [29]. For leaf samples collected immediately after treatment and incubated for various times, whitefly mortality and mycosis levels were analyzed with repeated measures with Proc Mixed procedure with block as a random factor; residual plots were checked for normality. For other samplings, immature mortality and mycosis levels were analyzed with Proc GLM using beta distribution and logit function; count data (live immatures, CFU) were analyzed with negative binomial distribution and log function. Means between treatments were separated by Tukey’s test at α = 0.05.

## 3. Results

### 3.1. Field Efficacy in 2019

**Figure 1:**
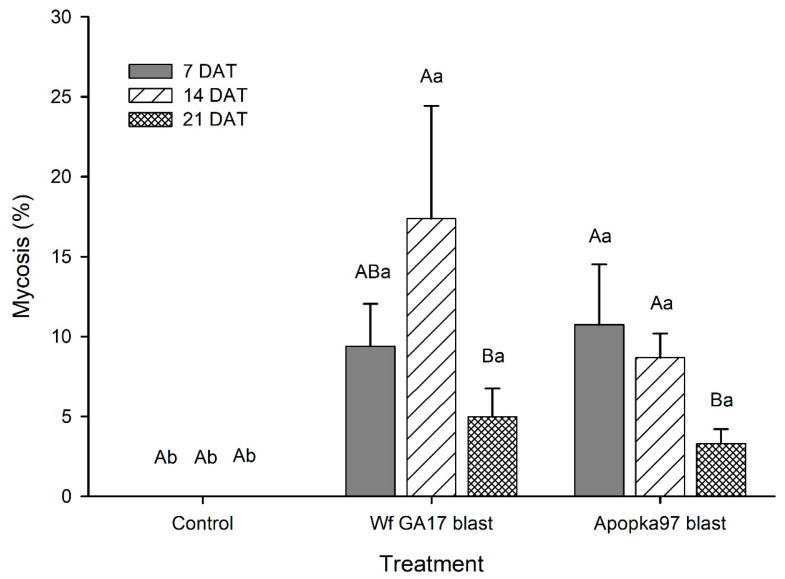
Percent mycosis in whitefly immatures treated by *Cordyceps javanica* Wf GA17 strain or Apopka97 strain blastospores (blast) for cotton leaf samples collected at 7, 14 and 21 days after treatment (DAT) in 2019 trials 1 and 2. The same capital and lower-case letters indicate no significant difference between times and between treatments, respectively (Tukey’s, α = 0.05).

In 2019 trials 1 and 2 (cotton), there were no significant interactions between treatment and trials (adults: F_2,78_ = 1.33, *p* = 0.2692; immatures: F_2,78_ = 1.42, *p* = 0.2471); thus, data from the two trials were combined for analysis. Prior to treatment, the number of whitefly adults ranged from 11 ± 2 to 15 ± 4 per leaf, and immatures ranged from 36 ± 8 to 38 ± 6 per leaf by averaging four leaf samples/plot. No treatment effects were detected in reducing the number of adults (F_2,78_ = 1.39, *p* = 0.2560) or immatures (F_2,78_ = 1.48, *p* = 0.2337); numbers of both adults (F_3,78_ = 21.75, *p* < 0.0001) and immatures (F_3,78_ = 12.20, *p* < 0.0001) declined over time regardless of treatments, with no significant interactions between treatment and sampling time (adults: F_6,78_ = 0.77, *p* = 0.5977; immatures: F_6,78_ = 1.21, *p* = 0.3081). Although whitefly numbers were not suppressed, blastospores of *C. javanica* Wf GA17 and Apopka97 strains caused significant and similar infection (up to 17%) (F_2,58_ = 31.89, *p* < 0.0001) (Figure 1). The mycosis level declined at 21 DAT for both fungal strains (F_2,58_ = 4.14, *p* = 0.0208). There was no significant interaction between treatment and time (F_4,58_ = 1.00, *p* = 0.4132).

### 3.2. Field Efficacy in 2020

#### 3.2.1. Laboratory Incubation of 0-Day Sampling

**Figure 2:**
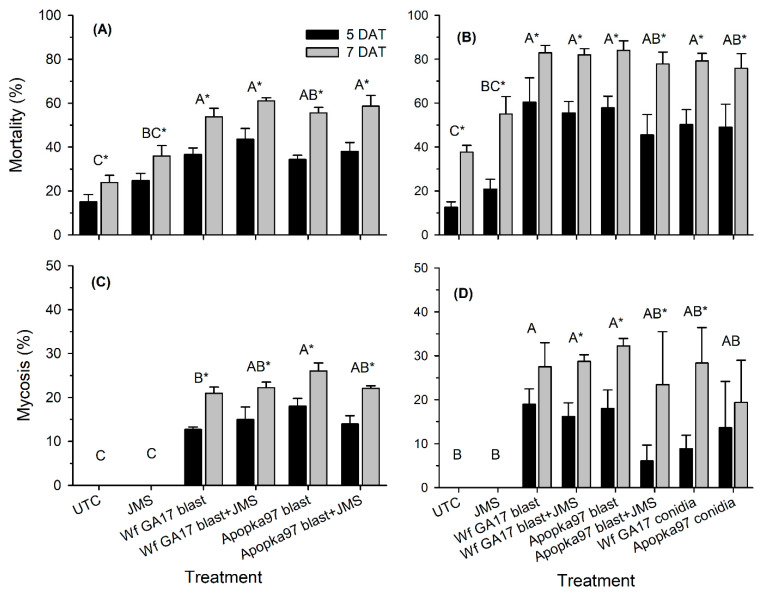
Mortality and percent mycosis in whitefly immatures treated by *Cordyceps javanica* Wf GA17 strain or Apopka97 strain blastospores (blast) alone (or conidia alone), combined with JMS stylet oil, or JMS oil alone for cotton leaves collected immediately after application and incubated at 25 °C for 5 and 7 days after treatment (DAT) in 2020 trial 1 (**A**,**C**) and trial 2 (**B**,**D**). The total number of whiteflies examined per plot ranged 48–165 in trial 1 and 5–29 in trial 2. Within each subgraph, the same letters suggest no significant difference between treatments with repeated measures; * indicates difference between observation time for each treatment (Tukey’s, α = 0.05).

In 2020, the JMS stylet oil was added to fungal treatments to improve control. In 2020 trial 1 (cotton), for leaf samples collected immediately after treatment and incubated for 5 and 7 days, whitefly immature mortality was significantly affected by treatment (F_5,33_ = 13.52, *p* < 0.0001), incubation time (F_1,33_ = 312.52, *p* < 0.0001) and their interaction (F_5,33_ = 5.14, *p* = 0.0014) (Figure 2A). Compared with the untreated control, all treatments except JMS oil alone caused significant mortality of whitefly immatures. *C. javanica* Wf GA17 strain blastospores alone and in combination with JMS oil and Apopka97 strain blastospores plus JMS oil had the highest mortality levels, significantly higher than JMS oil alone but not different from Apopka97 blastospores alone. Insect mortality increased from 5 to 7 DAT in all treatments. In mycosis occurrence, there were significant differences among treatments (F_5,33_ = 72.01, *p* < 0.0001) and between incubation times (F_1,33_ = 81.51, *p* < 0.0001), with significant interactions (F_5,33_ = 8.20, *p* < 0.0001) (Figure 2C). All fungal treatments caused significant infection in immatures. Apopka97 strain blastospores alone had the highest infection level, which was not different from JMS oil combined with Wf GA17 or Apopka97 blastospores but higher than Wf GA17 blastospores alone. Mycosis levels advanced from 5 to 7 DAT in all fungal treatments. No natural fungal infection was observed in control and JMS alone.

In 2020 trial 2 (cotton), conidial applications of both fungal strains alone were added. Immature mortality varied with treatment (F_7,45_ = 9.45, *p* < 0.0001) and incubation time (F_1,45_ = 216.79, *p* < 0.0001); there were no significant interactions (F_7,45_ = 0.53, *p* = 0.8108) (Figure 2B). The pattern was similar to trial 1, where all fungal treatments caused similar but significant levels of immature mortality. Apopka97 blastospores combined with JMS oil was not significantly different from the oil alone. There were no differences between blastospores and conidia of either fungal strain alone in causing immature mortality. Mycosis levels were affected by both treatment (F_7,45_ = 4.10, *p* = 0.0015) and incubation time (F_1,45_ = 32.46, *p* < 0.0001) and their interactions (F_7,45_ = 2.37, *p* = 0.0375) (Figure 2D). Wf GA17 blastospores alone and in combination with JMS oil and Apopka97 blastospores alone had the highest levels of mycosis occurrence. There were dramatic variations among plots in the two conidial applications and Apopka97 blastospores plus JMS oil, which did not have significant infection overall.

#### 3.2.2. Weekly Field Sampling Post-Treatment

**Figure 3:**
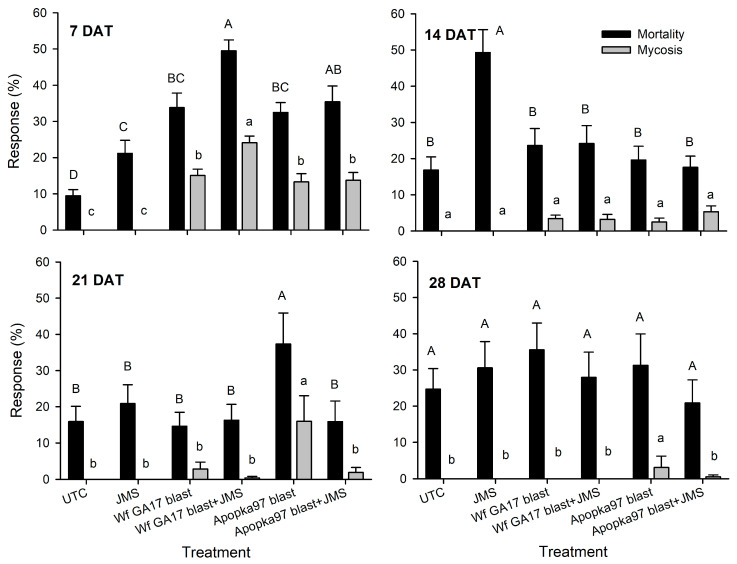
Mortality and percent mycosis in whitefly immatures treated by *Cordyceps javanica* Wf GA17 strain or Apopka97 strain blastospores (blast) alone or combined with JMS stylet oil or JMS oil alone for cotton leaves collected at 7, 14, 21 and 28 days after treatment (DAT) in 2020 trial 1. The same capital and lower-case letters indicate no significant difference between treatments in mortality and mycosis levels, respectively, in each subgraph (Tukey’s, α = 0.05).

Other than 0-day sampling, additional leaf samples were taken at various times after application to evaluate the field efficacy. In 2020 trial 1, at 7 DAT all treatments caused significant immature mortality compared with control (F_5,95_ = 13.84, *p* < 0.0001) (Figure 3). Wf GA17 blastospores combined with JMS oil induced the highest mortality, significantly higher than Wf GA17 or JMS alone. The combination of Apopka97 and JMS oil incurred higher mortality than JMS alone but was not different from the fungus alone. There were no differences between Wf GA17 and Apopka97 blastospores applied either alone or in combination with the oil. At 7 DAT, mycosis was observed in all fungal treatments, with the highest level found in the combination of Wf GA17 with oil (F_5,95_ = 8.68, *p* < 0.0001). At 14 DAT, significant immature mortality was only seen in JMS oil alone but not in any fungal treatment (F_5,95_ = 7.39, *p* < 0.0001); no significant mycosis was observed (F_5,95_ = 2.06, *p* = 0.0768). Noticeably, despite JMS oil alone caused higher mortality than the combination with Wf GA17 or Apopka97 blastospores at 14 DAT, the add-up mortality of 7 and 14 DAT was similar, and the combined treatments appeared to act faster than the oil alone. At 21 DAT, Apopka97 blastospores alone caused higher whitefly mortality (F_5,95_ = 2.73, *p* = 0.0237) and mycosis occurrence (F_5,95_ = 5.75, *p* = 0.0001) than other treatments. At 28 DAT, there were no treatment differences in immature mortality (F_5,85_ = 0.53, *p* = 0.7497), but Apopka97 alone appeared to cause mycosis although at a very low level (F_5,85_ = 9.36, *p* < 0.0001).

**Figure 4:**
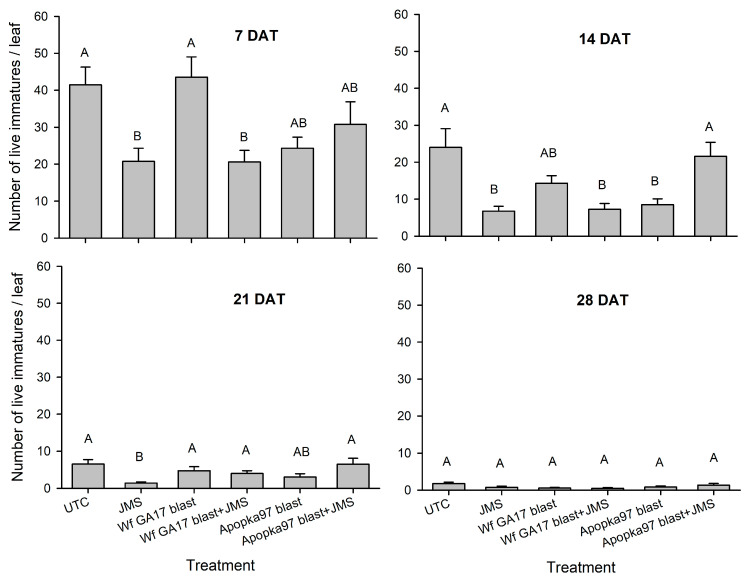
Number of live whitefly immatures on cotton leaves treated with *Cordyceps javanica* Wf GA17 strain or Apopka97 strain blastospores (blast) alone or combined with JMS stylet oil or JMS oil alone for leaf samples collected at 7, 14, 21 and 28 days after treatment (DAT) in 2020 trial 1. The same letters indicate no significant difference between treatments in each subgraph (Tukey’s, α = 0.05).

For live immature counts in 2020 trial 1, at 7 DAT JMS oil applied alone or in combination with Wf GA17 blastospores significantly reduced whitefly populations compared with control, but did not with Apopka97 alone or together with the oil (F_5,95_ = 4.91, *p* = 0.0005) (Figure 4). At 14 DAT, JMS alone and mixed with Wf GA17 continued to show efficacy; meanwhile, Apopka97 alone also reduced the number of whitefly immatures (F_5,95_ = 8.72, *p* < 0.0001). At 21 DAT, only JMS oil showed population decline (F_5,114_ = 5.27, *p* = 0.0002). At 28 DAT, no treatment suppressed whitefly populations (F_5,95_ = 2.30, *p* = 0.0508). During the test, whitefly populations declined over time (F_3,437_ = 210.02, *p* < 0.0001) across treatments (no interaction: F_15,437_ = 1.34, *p* = 0.1741). Crossing all sampling times, JMS alone, Wf GA17 plus JMS and Apopka97 alone were effective in lowering whitefly densities, while Wf GA17 alone and Apopka97 plus JMS were not (F_5,437_ = 16.65, *p* < 0.0001).

**Figure 5:**
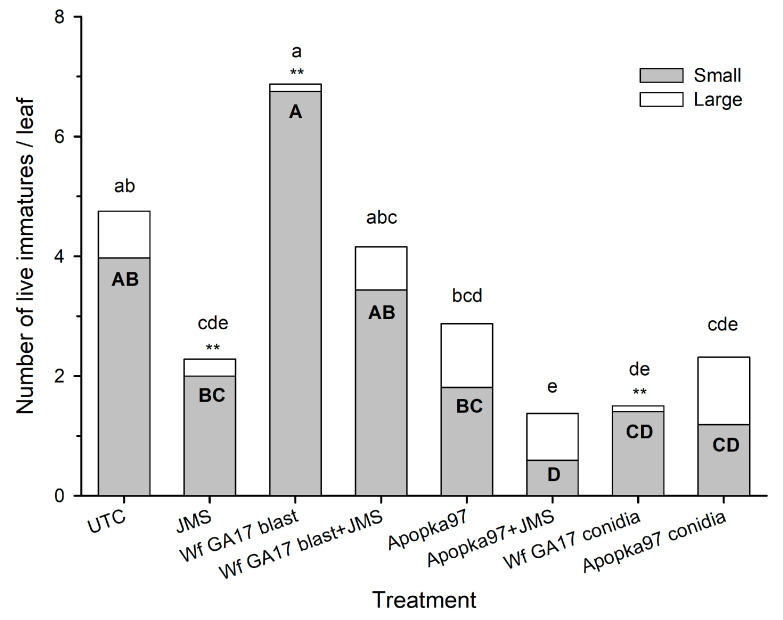
Number of small, large and total (small + large) live whitefly immatures on cotton leaves treated with *Cordyceps javanica* Wf GA17 strain or Apopka97 strain blastospores (blast) alone or combined with JMS stylet oil, conidia alone, or JMS oil alone for leaf samples collected at 7 days after treatment in 2020 trial 2. The same capital and lower-case letters indicate no significant difference between treatments for small and total counts, respectively; ** suggest difference from the untreated control for large immature counts (Tukey’s, α = 0.05).

In 2020 trial 2, there were low numbers of large immatures (3rd instar) and more small immatures (1st and 2nd instar) present in samplings, especially in the early phase. Small immature counts varied vastly with sampling time (F_3,989_ = 51.86, *p* < 0.0001) but did not differ with treatment (F_7,989_ = 1.47, *p* = 0.1736), which interacted significantly with sampling time (F_21,989_ = 3.17, *p* < 0.0001). Treatment effects only appeared at 7 DAT when Apopka97 blastospores + JMS and conidia of both fungal strains had fewer small immatures than control (4.0/leaf) (Figure 5). The number of large immatures fluctuated with treatment (F_7,989_ = 6.03, *p* < 0.0001) and sampling time (F_3,989_ = 103.69, *p* < 0.0001), with significant interactions (F_21,989_ = 2.07, *p* = 0.0031); but in all samples only at 7 DAT Wf GA17 blastospores and conidia and JMS alone had lower numbers than control plots (0.8/leaf), and immature mortality was not evaluated due to low numbers. Combining small and large immatures, both treatment (F_7,989_ = 2.92, *p* = 0.0049) and sampling time (F_3,989_ = 50.72, *p* < 0.0001) affected live counts with significant interactions (F_21,989_ = 3.19, *p* < 0.0001); in all samples only JMS alone and combined with Apopka97 blastospores, and conidia forms of both Apopka97 and Wf GA17 had fewer live immatures than control (4.8/leaf) at 7 DAT. Combining all eight leaves per plot, there was low immature mortality (ranged 0.0 ± 0.0% to 16.9 ± 6.5%) in treatments, not different from control in any sampling time (F_7,21_ ≤ 1.59, *p* ≥ 0.1919). Very few fungal infection was observed regardless of treatments (conidia or blastospores; Wf GA17 or Apopka97 with or without JMS oil).

**Figure 6:**
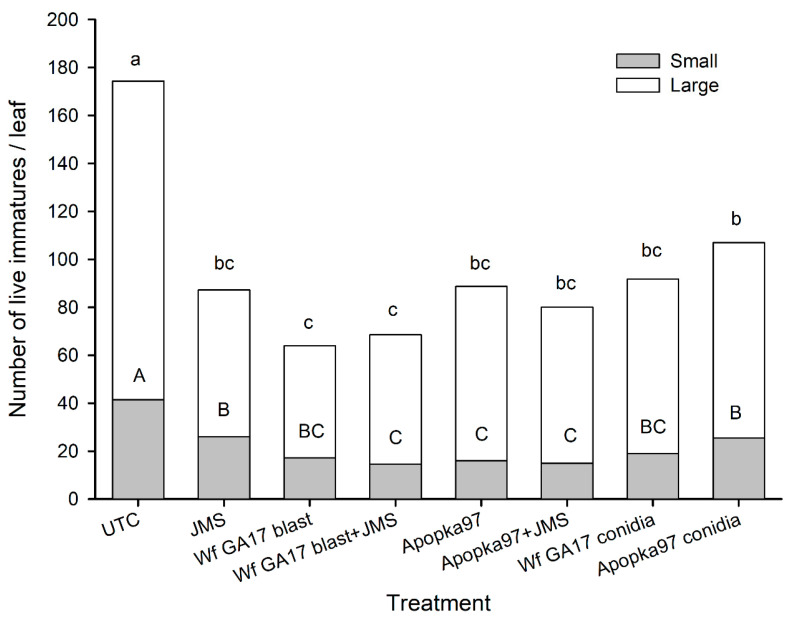
Number of small, large and total (small + large) live whitefly immatures on snap bean leaves treated with *Cordyceps javanica* Wf GA17 strain or Apopka97 strain blastospores (blast) alone or combined with JMS stylet oil, conidia alone, or JMS oil alone for leaf samples collected at 21 days after treatment in 2020 trial 3. The same capital and lower-case letters indicate no significant difference between treatments for small and total counts, respectively (Tukey’s, α = 0.05).

The same treatments were repeated in 2020 trial 3 (snap bean). Similar to trial 2, there were fewer large immatures and more small immatures at the beginning of the test. The number of small immatures varied with treatment (F_7,449_ = 5.50, *p* < 0.0001) and decreased with sampling time (F_2,449_ = 162.69, *p* < 0.0001); treatment differences only appeared at 21 DAT when all treatments had fewer small immatures than the control, with Wf GA17 blastospores + JMS, and Apopka97 blastospores alone or combined with JMS being the lowest (Figure 6). The number of large immatures increased with sampling time (F_2,449_ = 10.29, *p* < 0.0001) but was not affected by treatment (F_7,449_ = 0.85, *p* = 0.5490), although Wf GA17 blastospores alone had marginally lower number of large immatures than the control at 21 DAT. Adding up small and large immature counts, there were significant differences between treatments (F_7,449_ = 3.82, *p* = 0.0005) but not between observation time (F_2,449_ = 1.38, *p* = 0.2537); compared with control plots, all treatments had fewer live immatures, with Wf GA17 blastospores alone or combined with JMS being the lowest. There was no significant interaction between treatment and sampling time in the number of small and large immatures or combined (F_14,449_ ≤ 1.45, *p* ≥ 0.1276).

#### 3.2.3. Field Persistence of Fungal Propagules

**Figure 7:**
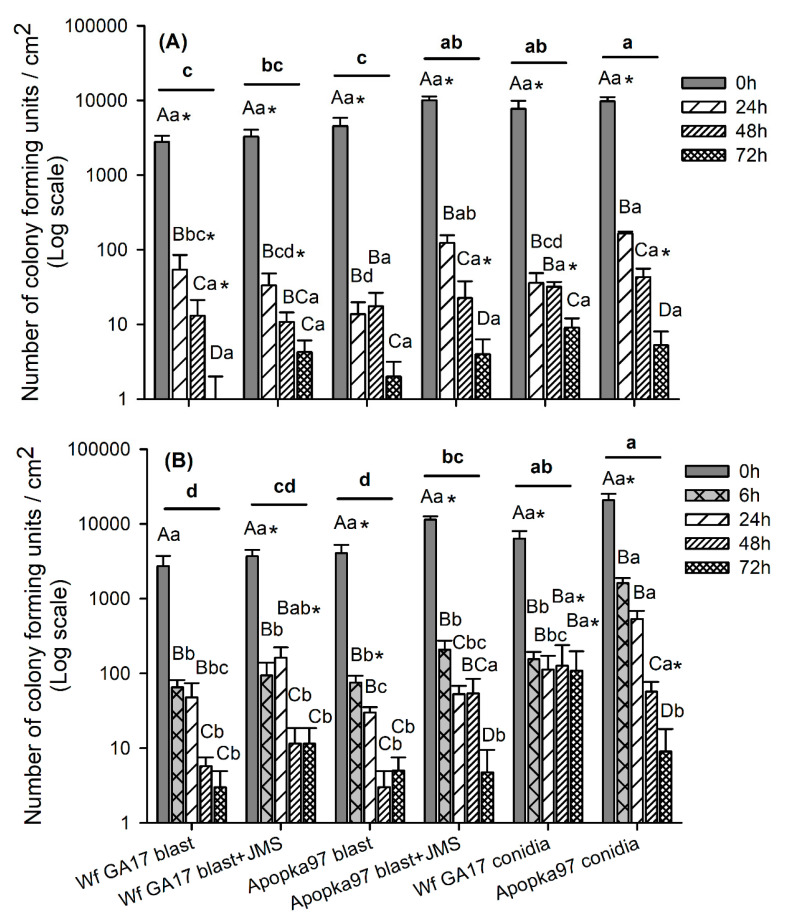
The number of colony forming units of fungi recovered from leaf surfaces (upper and lower surfaces combined) at various time after treatment with *Cordyceps javanica* Wf GA17 or Apopka97 strain blastospores (blast) alone or combined with JMS stylet oil, or conidia alone in 2020 trial 2 (cotton field-(**A**)) and trial 3 (snap bean field-(**B**)). The same capital and lower-case letters indicate no significant difference between times and between treatments, respectively, in each subgraph; asterisk suggest difference between upper and lower leaf surfaces; different bolded lower-case letters above lines suggest treatment differences crossing all sampling times (Tukey’s, α = 0.05).

In 2020 trial 2, fungal persistence was evaluated by the number of CFUs, which varied with leaf surface (F_1,144_ = 65.00, *p* < 0.0001), time after application (F_3,144_ = 385.23, *p* < 0.0001) and treatment (F_5,144_ = 9.74, *p* < 0.0001). There were significant interactions between surface and time (F_3,144_ = 21.23, *p* < 0.0001), between time and treatment (F_15,144_ = 2.36, *p* = 0.0047), and in surface × time × treatment (F_15,144_ = 1.83, *p* = 0.0363), but not between surface and treatment (F_5,144_ = 1.10, *p* = 0.3653). At 0 h, CFU counts recovered from the upper surface were 12–19 times of those from the lower surface, suggesting most spores deposited on the upper surface. There was a 98.3–99.8% decline in 24 h after treatment on the upper surface and a 86.2–98.2% decline on the lower leaf surface in all fungal treatments, regardless of fungal strain, propagule form (blastospores or conidia) and application method (alone or in combination with JMS). At ≥24 h after application, the difference between leaf surfaces was dramatically reduced, and there were very few CFUs recovered from either surface at 72 h evaluation regardless of treatments. Combining leaf surfaces, treatment differences only appeared at 24 h, when Apopka97 conidia had the most CFUs, higher than other treatments except Apopka97 blastospores mixed with JMS oil. Crossing evaluation times, there was no difference between the two fungal strains (conidia or blastospores, alone or combined with JMS), but conidia had better persistence than blastospores for both strains; Wf GA17 blastospores alone was similar to that mixed with JMS, while Apopka97 blastospores combined with JMS was higher than applied alone (time: F_3,72_ = 347.48, *p* < 0.0001; treatment: F_5,72_ = 7.04, *p* < 0.0001; time × treatment: F_15,72_ = 1.38, *p* = 0.1803) (Figure 7A).

In the repeated test in 2020 trial 3 (snap bean), 6 h post-application was added to the evaluation times. In CFU counts, there were significant effects of time after application (F_4,180_ = 284.42, *p* < 0.0001) and treatment (F_5,180_ = 19.11, *p* < 0.0001). However, different from the cotton field trial, leaf surface did not have a significant effect (F_1,180_ = 0.02, *p* = 0.8882), although it had significant interactions with time (surface × time: F_4,180_ = 15.34, *p* < 0.0001) and treatment (surface × treatment: F_5,180_ = 3.27, *p* = 0.0076); there were significant interactions between time and treatment (F_20,180_ = 3.22, *p* < 0.0001) but not in surface × treatment × time (F_20,180_ = 1.46, *p* = 0.1006). At 0 h, the number of CFUs on upper surface was 4–8 times of that on lower surface except that Apopka97 blastospores + JMS was 17 times. At 6 h after application, CFU numbers declined dramatically (97.8–99.7% on upper leaf surface and 49.8–93.2% on the lower surface). In ≥6 h samplings, CFU numbers on both leaf surfaces were similar in most treatments, and a few observations (Wf GA17 conidia at 48–72 h and Apopka97 conidia at 48 h) had even more CFUs on the lower surface. Combining leaf surfaces, Apopka97 conidia had the highest number of CFUs at 6–24 h, and Wf GA17 conidia was the highest at 48–72 h; in particular, Wf GA17 conidia had no further decline from 6 h to 72 h. Across evaluation times, it followed a very similar pattern as the cotton field test that the two fungal strains had similar persistence regardless of propagule form (conidia or blastospores) and application method (alone or combined with JMS), conidia consistently persisted better than blastospores, and JMS oil enhanced persistence of Apopka97 but not of Wf GA17 compared to blastospores alone (time: F_4,90_ = 155.65, *p* < 0.0001; treatment: F_5,90_ = 17.21, *p* < 0.0001; time × treatment: F_20,90_ = 2.72, *p* = 0.0007) (Figure 7B).

## 4. Discussion

Previously, laboratory tests showed superior virulence of *C. javanica* Wf GA17 against whiteflies [7]. Here, results under field conditions suggest that the efficacy of Wf GA17 was not different from the commercial Apopka97 strain. In 2019 field trials, neither fungus provided significant control and the infection levels were very low (below 20% maximum), when fungal blastospores applied alone. In 2020 trials, when leaf samples were collected immediately following application (minimum outdoor exposure to environmental conditions), both fungal strains applied alone or mixed with JMS oil achieved considerate immature mortality. Nonetheless, in follow-up field samplings, whiteflies were mostly killed within 7 days. Among treatments, the combination of Wf GA17 blastospores and JMS oil appeared to be most effective, which was consistently shown in 2020 trial 1 (cotton) and trial 3 (snap bean). However, the pattern was different in 2020 trial 2 (cotton), in which Apopka97 blastospores combined with JMS was most suppressive to whiteflies, while Wf GA17 blastospores + JMS seemed to be ineffective. It was observed that JMS oil alone caused significant control and reduced whitefly populations, as shown in all three field trials in 2020. When fungal blastospores applied alone, the efficacy of Wf GA17 and Apopka97 fluctuated with trials; they reduced whitefly numbers in some trials (e.g., 2020 trial 3) but were ineffective in others. The difference between blastospores applied alone and in combination with JMS only appeared for Wf GA17 in 2020 trial 1 and Apopka97 in 2020 trial 2.

The authors hypothesize that the inconsistent and low field efficacy of fungi were due to short fungal persistence. This hypothesis was verified in later persistence tests, in which most spores lost viability within 24 h (2020 trial 2) and even as short as 6 h (2020 trial 3) post-application regardless of propagule form (conidia or blastospores) and application method (alone or combined with JMS). Undoubtedly, the rapid decline in viability of fungal propagules dramatically reduced the activity of fungi in effective infection of whiteflies. Environmental factors, such as temperature, solar radiation, humidity and rainfall play a significant role in EPF persistence and control success [2,3,4,5]. High temperature is detrimental to fungal spores and may reduce the activity and infection, as shown in the previous report that the virulence of *C. javanica* Wf GA17 was greatly reduced at above 30 °C and at ≥35 °C most conidia lost viability in short time [9]. The weather was hot and dry in 2019 during test periods, especially in trial 2 where there was only 0.1 mm rain in total; in both trials there were high temperatures (close to 35 °C or above), strong solar radiation and no rain in several days following application (although trial 1 had rain afterwards), which adversely affected the survival and efficacy of both fungal strains. In 2020, environmental conditions during the first cotton test were relatively mild and the maximum temperature did not exceed 35 °C; in particular, there were frequent rainfalls, weak solar radiation and mild temperature (below 30 °C) in several days post-treatment application, which might have created an environment suitable for fungal activities, although rain might have also washed spores off leaf substrates, reducing propagules that could have taken effect. In 2020 trials 2 and 3 (conducted in October–November, 2020), the temperature dropped and solar radiation was weaker; still, the conditions were dry, which combined with other unknown factors might have compromised the fungal efficacy.

Interestingly, in the snap bean test (2020 trial 3) treatment effects did not show until at 21 DAT. Additionally, later phase infections were observed at 21 and 28 DAT in 2020 trial 1, despite at low levels. This was probably because it took longer for fungal propagules to be effective under low temperatures, or spores became dormant for a period of time and resumed activities when conditions were favorable, or the fungus propagated on hosts to reach a higher level to cause infection. Pick et al. [30] found that the number of CFUs of *C. javanica* Apopka97 (*I. fumosorosea* of PFR-97™) declined at 7 days post-treatment compared with 0-day in weekly samples of field-caged citrus leaves but increased after 35 days, and there was a residue effect in causing mortality (up to 40%) of the Asian citrus psyllid, *Diaphorina citri* Kuwayama, over a 35-day experimental period. Conversely, Avery et al. [31] reported that in the field assessment of PFR-97™ against the invasive ficus whitefly, *Singhiella simplex* (Singh), the CFU number increased from day 1 to day 7 post-treatment and then declined. It was suspected that the increased fungal activity was associated with germination of blastospores on leaf surfaces under favorable environmental conditions in Florida, and possibly also biotic factors such as arthropod hosts, while declined CFUs in other observations might be due to rainfall, biodegradation or other factors.

Blastospores differ greatly from conidia in structure and properties. Blastospores are thin-walled and hydrophilic whereas conidia have thicker walls and are hydrophobic. Blastospores are vegetative propagules growing in the haemocoel of infected hosts for many EPF including *C. javanica*, while conidium is the most common type of propagule occurring naturally; the former germinate faster and are more virulent than conidia, but because of structural differences they are also more vulnerable to environmental stresses and less stable [32,33,34,35]. For example, blastospores of *I. fumosorosea* SFP-198 were less thermotolerant than conidia and the addition of corn oil enhanced the thermotolerance [36]. In this study, for both *C. javanica* Wf GA17 and Apopka97 strains, conidia consistently persisted better than blastospores applied alone and reduced whitefly populations, as shown in both field persistence tests (2020 trials 2 and 3). However, there was a similarly high reduction in CFUs to be received by target hosts, and the two strains were not different in field persistence regardless of propagule form (conidia or blastospores) and application method (alone or combined with JMS) in both trials. Additionally, it was consistently shown that JMS oil improved persistence of Apopka97 blastospores but not Wf GA17. This effect was rather limited as most blastospores still lost activity rapidly even with the addition of oil. In terms of efficacy, Wf GA17 conidia were more effective than blastospores in reducing whiteflies in 2020 trial 2 but did not work similarly in 2020 trial 3; such a difference between propagules did not occur in Apopka97.

In addition to short persistence caused by environmental stress, lack of adequate coverage might be another reason explaining for the limited control efficacy. The mode of action of EPF requires spore adhesion to insect cuticles via direct contact to initiate the infection process, followed by the formation of appressoria penetrating the insect cuticle, mycelial growth that kills the host, and the production of asexual conidia that disperse to start the next infection cycle [2]. Most spores (80–90%) were recovered from the upper surface of cotton leaves, while whitefly immatures develop on the lower leaf surface. Hence, most spores applied to leaf substrates lacked adequate contact with the target whiteflies and were ineffective in killing the pest. Despite strong evidence that most spores landed on upper leaf surface, after only a few hours there were more viable spores on the lower leaf surface, strongly suggesting that spores lost viability soon after application and those delivered to the lower leaf surface were protected from direct sunlight exposure and persisted longer, consistent with the report of Jaronski [3]. In snap bean, there was no difference between leaf surfaces in spore persistence, which is expected because snap bean leaves are not parallel with the ground as with cotton leaves. To improve control efficacy, it is critical to leverage spore coverage and dispersion. Spores in the tests were delivered using a 4-nozzle bloom sprayer. Other types of sprayers, such as electrostatic and air blast sprayers with better coverage may be considered for future tests.

Given the short persistence of *C. javanica* in the field, multiple applications and/or increased rate may be desirable to boost the control levels, especially for new growths of pests that were not exposed to fungi in previous sprays. Wraight et al. [23] reported that in small-scale field trials, multiple applications of unformulated conidia of *P. fumosoroseus* and *B. bassiana* made at intervals of 4–5 days at 5 × 10^13^ conidia/ha using an electrostatic air-assist sprayer achieved over 90% control of large nymphs of *B. argentifolii* (*B. tabaci*) on cucumbers and cantaloupe melons, and those applied at 1.25 × 10^13^ conidia/ha at 4- to 5-day intervals reduced numbers of large nymphs by 85% in cantaloupe, although the fungi were ineffective against whitefly adults. Similarly, Jaronski and Lord [37] found that multiple applications of *B. bassiana* GHA at 2.5 × 10^13^ conidia/ha in a weekly interval provided over 70% control of *B. argentifolii* nymphs in an irrigated cantaloupe field in southern California. Additionally, Ou et al. [24] reported 55% mortality of *B. tabaci* caused by *C. javanica* at 1 × 10^8^ conidia/mL in a semi-field (cage) study at 15 days post-application in cotton, but the application rate was approximately 5 times of the label rate of similar products (PFR-97). This research mostly used the rate of 5 × 10^12^ viable spores/ha (except 2019 trial 1 used 2.5 × 10^12^ viable spores/ha and 2020 trial 3 used 1 × 10^13^ viable spores/ha) in a single application. The lower rate and lack of repeated applications might have at least partially accounted for the inconsistent and low control levels. However, given that the used rates were close to the label rate of PFR-97, undoubtedly, increasing rate and application times would increase the material costs, while both efficacy and costs are essential considerations in strategic pest management that requires optimum control with affordable costs.

## 5. Conclusions

Overall, *C. javanica* Wf GA17 and Apopka97 strains acted similarly in field trials against whiteflies, irrespective of application methods (alone or combined with JMS oil) and propagule forms (blastospores or conidia). However, neither *C. javanica* Wf GA17 nor Apopka97 provided satisfactory control when applied alone, but Wf GA17 blastospores combined with JMS oil achieved higher control levels in some tests. The two fungal strains exhibited similar field persistence that most spores lost viability shortly after application in the field regardless of application method (alone or mixed with JMS), but conidia persisted better than blastospores for both strains. The short fungal persistence and lack of adequate coverage might have accounted for inconsistent and low control efficacy. Future studies will be directed to formulation development and enhancing spray coverage to improve spore persistence and efficacy levels in the open field and controlled environment.

## Data Availability

Data stored at the USDA-ARS, SE Fruit and Tree Nut Research Station may be made available upon request.
